# Pharyngeal Packing during Rhinoplasty: Advantages and Disadvantages

**Published:** 2015-11

**Authors:** Majid Razavi, Mehryar Taghavi Gilani, Ali Reza Bameshki, Reza Behdani, Ehsan Khadivi, Mahdi Bakhshaee

**Affiliations:** 1 *Cardiac Anesthesia Research Center, Imam-Reza Hospital, Faculty of Medicine, Mashhad University of Medical Science, Mashhad, Iran. *; 2 *General Practitioner, Young Researchers Club and elites, Mashhad Branch, Islamic Azad University, Mashhad, Iran.*; 3 *Sinus and Surgical Endoscopic Research Center, GhaemHospital, Faculty of Medicine, Mashhad University of Medical Sciences, Mashhad, Iran.*

**Keywords:** Nausea and vomiting, Pharyngeal pack, Sore throat, Septorhinoplasty

## Abstract

**Introduction:**

Controversy remains as to the advantages and disadvantages of pharyngeal packing during septorhinoplasty. Our study investigated the effect of pharyngeal packing on postoperative nausea and vomiting and sore throat following this type of surgery or septorhinoplasty.

**Materials and Methods:**

This clinical trial was performed on 90 American Society of Anesthesiologists (ASA) I or II patients who were candidates for septorhinoplasty. They were randomly divided into two groups. Patients in the study group had received pharyngeal packing while those in the control group had not. The incidence of nausea and vomiting and sore throat based on the visual analog scale (VAS) was evaluated postoperatively in the recovery room as well as at 2, 6 and 24 hours.

**Results:**

The incidence of postoperative nausea and vomiting (PONV) was 12.3%, with no significant difference between the study and control groups. Sore throat was reported in 50.5% of cases overall (56.8% on pack group and 44.4% on control). Although the severity of pain was higher in the study group at all times, the incidence in the two groups did not differ significantly.

**Conclusion:**

The use of pharyngeal packing has no effect in reducing the incidence of nausea and vomiting and sore throat after surgery. Given that induced hypotension is used as the routine method of anesthesia in septorhinoplasty surgery, with a low incidence of hemorrhage and a high risk of unintended retention of pharyngeal packing, its routine use is not recommended for this procedure.

## Introduction

Throat pain, nausea and vomiting are some of the most common complications following surgery and anesthesia. Such complications have economic implications and may lead to reduced functioning and a sense of discomfort after surgery (1). As blood is a potent emetic, any major postoperative nausea and vomiting (PONV) in the immediate postoperative period may result in aspiration of gastric contents. Accordingly, one of the main factors in patient satisfaction following a surgical operation is the absence of nausea, vomiting and sore throat (1,2). Due to the higher risk of blood ingestion in nasal surgery, oral and pharyngeal packing is used to minimize the risk of vomiting (3,4). 

Several studies found that placing packing in the retropharyngeal space after intubation absorbs most of the blood loss and reduces the risk of aspiration of blood and bone and tissue fragments into the airway and digestive tract, leading to a lower incidence of nausea and vomiting (4). However, it has been seen that packing does not provide full protection and that the patient can still experience a sore throat(2,3), trauma and edema in oral and pharyngeal structures after surgery (2-4). There is also the risk of leaving the pack inadvertently in place after extubation which can lead to airway obstruction and intestinal occlusion as well as complications such as oral aphthosisand acute tongue enlargement (5-7). The prevalence of sore throat following intubation varies from 14.4% to 50% (1) and has been reported in up to 60% of cases when pharyngeal packs were applied (8). Therefore, some debate remains as to whether or not they should be used in medical centers. In some countries such as Great Britain they are used routinely, whereas in North America their application is quite limited (5).

This study aimed to investigate the effect of pharyngeal packing on the incidence of PONV and sore throat in patients having undergone septorhinoplasty surgery.

## Materials and Methods

In a prospective clinical trial, 90 patients referred to the ear, nose and throat (ENT) clinic in the Imam Reza Educational Hospital, Mashhad, Iran between Feb 2012 and May 2013 who were candidates for septorhinoplasty surgery, were entered into this study. Patients were randomly allocated into two groups: the study group (group A) with the placement of pharyngeal packing and the control group (group B) without such packs.

The patients were classified as American Society of Anesthesiologists (ASA) grade I or II, had no history of recent flu (in the past 2 weeks), coagulation disorders, motion sickness, difficult intubation (more than one laryngoscope or intubation attempt) or drug addiction. One patient was excluded from the study due to intubation problems, resulting in 44 and 45 patients in the study and control groups, respectively.

The study protocol was approved by the Research Council Ethics Committee of Mashhad University of Medical Sciences. Informed consent was obtained from each patient prior to entering the study.

Patients received no analgesic or sedative medication prior to surgery. At the point of recruitment into the study, patients were educated about postoperative throat pain versus nasal pain. Anesthetic induction was by fentanyl 2µg/kg, midazolam 2mg, and propofol 3mg/kg. Anesthesia was maintained by propofol infusion 50–100 µg/kg/min and remifentanil 0.1–0.5 µg/kg/min. During surgery all patients were given a single dose of ondansetron 4mg intravenously.

After inserting the correct (and same) size tracheal tube, a simple 16-ply thread gauze, 10×10cm in size, soaked in normal saline solution was placed into the pharyngeal space in the study group cases. All tracheal intubations were performed by an expert anesthesiologist. Once surgery had been completed and before moving the patient to recovery, the gauze was gently removed by an anesthesiologist, whilst the recovery unit nursing staff were blind to the group allocation. All patients were extubated in the recovery unit once fully awake. The presence of nausea and vomiting and the severity of sore throat based on the visual analog scale (VAS) scoring system (0 no pain to 10 severe pain) was recorded in the recovery room once patients were fully conscious in the recovery room and at 2,6 and 24 hours postoperatively. In order to evaluate the severity of throat pain more precisely, severity of sore throat was categorized into four classifications based upon the following qualitative indices:No pain (0), mild pain (1-3), moderate pain (4–7) and severe pain (>7). In the moderate pain group, a non-opioid sedative was administered and in the severe pain group intramuscular morphine was used after surgery. Confounding factors such as comorbidities and duration of anesthesia were also studied. Moreover, quantitative data were compared between the two groups using either the Student’s t-test or the Mann-Whitney U test. The Chi-square and Fisher’s exact test were applied to analyze qualitative data. SPSS version 16 was used for data analyses, with P<0.05 considered statistically significant.

## Results

Demographic data including age, sex, duration of anesthesia, and comorbidities were matched for the two groups (Table.1). Comorbidities were (with the exception of those resulting in exclusion from the study) classified into four categories: recent flu, seasonal allergy, asthma and hypertension. There was no statistically significant difference between the two experimental groups in terms of the incidence of these comorbidities (P=0.83) (Table. 1).

**Table 1:** The Demographic data of the Patients enrolled in the Study (mean±SD).

**Parameter**		**Group A (n=44)**	**Group B (n=45)**	**P value**
Age(y)		27.02	27.18±7.08	0.77
Gender(F/M)		39.5	40.5	1
Duration of anesthesia (min)		169.09±29.71	172.89±37.45	0.68
Co-existing disease (%)	Old common cold	1.1	1.1	0.83
	allergy	2.2	4.5	
	Asthma	1.1	0	
	hypertension	0	1.1	

The overall incidence of PONV was 12.3% (n=11). Eleven patients (12.3%) complained of post-surgical nausea; seven cases from the study group and four from the control group. In addition, three patients experienced post-surgical vomiting; one in the study group and two in the control group. No significant difference was observed between the two groups regarding PONV incidence (Table. 2).

**Table 2:** Incidence of postoperative nausea and vomiting N (%).

**Parameter**	**variable**	**GroupA(n=44)**	**GroupB(n=45)**	**Pvalue**
Nausea	Recovery room 2^nd^ h 6^th^ h 24^th^ h	3(6.8%) 3(6.8%) 0 1(2.3%)	1(2.2%) 2(4.4%) 0 1(2.2%)	0.36 0.67 0 1.0
Vomiting	Recovery room 2^nd^ h 6^th^ h 24^th^ h	0 0 0 1(2.3%)	1(2.2%) 1(2.2%) 0 0	1.0 0.49 0 0.49

Postoperative sore throat was recorded in 45 patients (50.5%); 25 in the study group and 20 in the control group. The incidence of sore throat categorized as mild, moderate in the recovery room and severe at 2,4, and 24 hours post surgery (recovery time) is shown in (Table.3). No statistically significant difference was seen between the two experimental groups in this regard. Three patients (3.3%) in the study group versus one in the control group required opioid analgesics in addition to non-steroidal analgesics for severe pain. However, there were no significant differences with opioid used in the incidence of PONV between the two groups.

**Table 3:** Distribution of sore throat

**Time**	**Pain**	**Study group (n=44)**	**Control group (n=45)**	**P value**
Recovery room	No Mild Moderate severe	27(61.4%) 5(11.4%) 10(22.7%) 2(4.5%)	33(73.3%) 5(11.2%) 6(13.3%) 1(2.2%)	0.61
2^nd^ hour	No Mild Moderate severe	30(68.2%) 5(11.4%) 6(13.6%) 3(6.8%)	37(82.2%) 5(11.1%) 3(6.7%) 0(0.0)	0.18
6^th^ hour	No Mild Moderate severe	37(84.1%) 2(4.5%) 4(9.1%) 1(2.3%)	40(88.9%) 0(0.0) 5(11.1%) 0(0.0)	0.44
24^th^ hour	No Mild Moderate severe	30(68.3%) 2(4.5%) 10(22.7%) 2(4.5%)	35(77.8%) 3(6.7%) 6(13.3%) 1(2.2%)	0.64

## Discussion

In the present study, the incidence of PONV showed no significant difference between the two experimental groups. Therefore, it could be concluded that factors other than blood ingestion play a major role in PONV in such patients; as reported by Kurkut and by Vanderberg et al (9,10). No significant difference in the prevalence of post-surgical sore throat was observed between the two groups, similar to the findings of Tay and Piltcher (11,12). However, as shown in Table 3, the number of patients with severe throat pain was significantly higher in the group using pharyngeal gauze versus controls at all timepoints studied. In addition, the need for opioid analgesics was also higher in this group (3.3% vs 1.1%).

The use of pharyngeal gauze in surgery carried out under general anesthesia, with a high risk of blood, other fluid or particle aspiration, is routine in some medical centers. Traditionally, woven gauze has been used, although today polyurethane foam is also employed in such procedures. 

Taking into account risks such as airway obstruction due to leaving the pack inadvertently in place, certain protocols have been designed to ensure the complete removal of surgical gauzes at the end of the operation (13,14). For this reason, they are now less frequently used in Britain compared to previous times, and their use has also been limited in North America (3,4).

There is an ongoing debate as to whether surgeons or anesthesiologists have overall responsibility for the removal of any packing used during surgery (5,13,14). In our center, since packing is applied by an anesthesiologist, the anesthesiologist is also considered responsible for its complete removal. PONV is associated with various factors such as underlying disease (motion sickness), female gender, smoking, method of anesthesia, type of drugs used and also palliative opioids. Blood ingestion is also a strong emetic factor (9,15). Sore throat following general anesthesia is usually related to the size of the intubation tube, laryngoscope and the passage of the nasogastric tube (9).

In research carried out prior to 2009, Basha et al. concluded that pharyngeal packing is associated with a more severe sore throat and a higher incidence of PONV after surgery (3), whereas other studies have found no change in the incidence of PONV and postoperative sore throat when pharyngeal packs were used compared with when they were not (11,12). A review article published in 2009 found no improvement in PONV with the use of pharyngeal packing and highlighted one study where greater throat pain was reported in those patients with pharyngeal packs compared with those without. Further studies focusing on certain nasal surgery are needed (8).

The majority of previous studies have addressed nasal surgery in general and have not focused on one particular type of surgery. In research by Korkut et al, however, nasal surgery was categorized. This study showed that the placement of pharyngeal packing had no effect on the incidence of PONV. It was also noted that hemorrhage during nasal surgery did not affect PONV and pharyngeal packs did not reduce blood ingestion, tracheal contamination, or both (9).

The higher rate of PONV in nasal surgery is thought to be due to the naso-emitic trigeminal-vagal reflex. In head and neck surgery the vagal nuclei in the brainstem are stimulated, which can result in vomiting (10).The etiology of sore throat following pharyngeal packing is due to the localized trauma and inflammation of the pharyngeal mucosa (16). On this basis, in order to reduce the incidence of trauma, different techniques have been used. In Marais et al. the use of a tampon was compared to pharyngeal gauze. They reported complaints of post-surgical sore throat in 38% of the patients receiving pharyngeal gauze compared with 15% of the tampon group (17). A study comparing three groups of patients with dry and wet pharyngeal gauzes and controls with no gauze showed no significant difference in the incidence rate of nausea, vomiting and sore throat following surgery (4).

In another study, esophageal packing was soaked with tenoxicam, an non-steroidal anti-inflammatorydrug (NSAID). The authors found that such patients experienced significantly less throat soreness after surgery (16). 

In the present study, comparisons between the two groups were limited to septorhinoplasty surgery. When induced hypotension was used as the anesthetic induction method, hemorrhage during the surgical procedure was highly reduced. In addition, due to the use of a tracheal tube cuff, the likelihood of blood ingestion or pulmonary aspiration is very low in this type of surgery. However, the need remains for studies which investigate the effect of intraoperative blood loss and hemodynamic status on the incidence of PONV. 

In our center, simple thread gauzes soaked in normal saline are routinely used; whereas based on the study by Marais et al, the application of tampons is likely to be associated with less throat pain (17). Nevertheless, in a 24-hour follow-up period, complications such as aphthous stomatitis did not occur. None of the patients in this study suffered sufficient pain or PONV to delay their discharge from hospital.One potential limitation of this study was the assessment of bleeding during surgery as a confounding factor. However, in this randomized trial, the effect of this factor on our results was limited. Future studies that consider bleeding record are recommended to further inform clinical practice. 

## Conclusion

Our findings showed that the use of pharyngeal packs in septorhinoplasty surgery did not affect the incidence of PONV and sore throat after surgery. However, the application of such gauzes follows a strict protocol so that major complications resulting from such packs being inadvertently left in place do not occur. With advances in general anesthesia, techniques such as induced hypotension are often used, resulting in a decreased risk of hemorrhage and associated reduced risk of blood ingestion or aspiration. Therefore, taking into account the clinical risk associated with packs being left in place after surgery, their routine use in septorhinoplasty is not recommended. However, further studies focusing on the degree of blood loss and the hemodynamic status of the patient during surgery with regards to their effects on PONV are needed. 
